# A gene trap mutagenesis screen for genes underlying cellular response to the mood stabilizer lithium

**DOI:** 10.1111/jcmm.12048

**Published:** 2013-04-12

**Authors:** Matthew Gow, Dora Mirembe, Zaomba Longwe, Benjamin S Pickard

**Affiliations:** a Undergraduate Biomedical Sciences Honours Degree Programmes, University of StrathclydeGlasgow, UK; b Strathclyde Institute of Pharmacy and Biomedical Sciences, University of StrathclydeGlasgow, UK; c CeNsUS: Centre for Neuroscience, University of StrathclydeGlasgow, UK

**Keywords:** gene trap, mutagenesis, lithium, mood stabilizer, screen, selection

## Abstract

Identifying the biological pathways mediating the action of a therapeutic compound may help the development of more specific treatments while also increasing our understanding of the underlying disease pathology. Salts of the metal lithium are commonly used as a front-line mood stabilizing treatment for bipolar disorder. Lithium's action has been variously linked to inositol phosphate metabolism and the WNT/Glycogen Synthase Kinase 3β (GSK3β)/β-Catenin signalling cascade, but, to date, little is known about which of these provides the principal therapeutic benefit for patients and, more specifically, which constituent genes, through presumed sequence variation, determine differences in patient response to treatment. Here, we describe a functional screen in which SH-SY5Y neuroblastoma cells were randomly mutated through genomic integration of the pMS1 poly A ‘gene trap’ plasmid vector. Lithium normally induces differentiation of neuroblastoma cells, but a small proportion of mutated cells continued to proliferate and formed colonies. Rapid amplification of cDNA ends (RACE)-PCR was used to identify the ‘trapped’ gene in each of these lithium-resistant colonies. Heterozygous, gene trap integrations were identified within ten genes, eight of which are likely to produce loss-of-function mutations including *MED10*, *MSI2* and three long intergenic non-coding (LINC) RNAs. Both *MED10* and *MSI2* have been previously linked with WNT/GSK3β/β-Catenin pathway function suggesting that this is an important mediator of lithium action in this screen. The methodology applied here provides a rapid, objective and economic approach to define the genetic contribution to drug action, but could also be readily adapted to any desired *in vitro* functional selection/screening paradigm.

## Introduction

Mutation screens in unicellular organisms such as bacteria and yeast have proved a valuable approach to dissect biological processes. They have also been applied to metazoans such as *Caenorhabditis elegans*, *Drosophila melanogaster*, *Danio rerio* and *Mus musculus*. The unicellular approach is quick, economically attractive and permits a wide range of selection criteria. In contrast, screens of higher organisms offer a more accurate model of human development, physiology and disease—but with increasing costs, time, use of animals and a more restricted repertoire of screening criteria.

Human laboratory cell lines are frequently used as tools to measure specific responses to drug treatments or to characterize specific gene mutations. However, they may also offer a useful ‘middle ground’ for primary mutation screens. One cell-based mutation approach has used RNAi libraries to knock down gene expression to expose specific cellular phenotypes. Some issues with this approach have been recently described, mostly concerning the specificity and efficiency of gene inhibition 1, 2. Gene trapping is a potential alternative approach to cellular mutagenesis that has been in existence for 25 years 3, 4. In common to all its varied manifestations is the inclusion of splice donor and/or splice acceptor sequences within the mutagenic plasmid constructs. After transfection/electroporation of the gene trap construct into a cell it stably integrates into the nuclear genomic DNA. If integration occurs within a gene intron then the splice acceptor/donor sequences divert and block the normal processes of exon–exon splicing that create the mature and complete mRNA from the gene's pre-mRNA. This has three consequences. Firstly, mRNA from the trapped allele of the gene is generally truncated or unstable resulting in a loss-of-function mutation. Secondly, the endogenous gene and gene trap sequences produce hybrid mRNAs with specific engineered properties. For example, β-galactosidase (β-gal) activity can be incorporated to reveal endogenous protein expression/localization, or an antibiotic resistance gene (*e.g*. Neo^R^: Neomycin/G418 resistance) to permit selection of productive gene trap events. Thirdly, these hybrid mRNAs comprising endogenous and gene trap-derived sequences can be readily amplified by rapid amplification of cDNA ends (RACE)-PCR protocols to identify the gene that has been trapped. To date, mammalian gene traps have been most commonly used to mutate mouse embryonic stem cells which are then used to generate mouse strains for large-scale phenotypic screens 5.

Here, we describe how pMS1, a gene trap vector of the ‘poly A’ type 6 was used to mutate the commonly used SH-SY5Y neuroblastoma cell line. The resulting population of mutant cells was subjected to an environmental selection pressure to identify cells with an acquired phenotypic response relevant to an *in vivo* biological question. In this instance we were interested in uncovering the biological processes affected by the mood stabilizer lithium in the context of its use for the treatment of the psychiatric illness bipolar disorder. Lithium is currently thought to offer neurotrophic or neuroprotective properties through the inhibition of glycogen synthase kinase 3β (GSK3β), a component of the WNT signalling pathway 7–10. This inhibition results in the intracellular accumulation of the downstream effector, β-Catenin which activates expression of neuroprotective genes such as *BCL-2*. Lithium has also been shown to affect the inositol pathway, specifically preventing inositol recycling by its inhibitory action on both inositol-1,4 bisphosphate 1-phosphatase and inositol-1-monophosphatase 11. Inositol(1,4,5)-trisphosphate (IP3), produced from inositol recycling, is crucial to neuronal function, controlling both gene expression and calcium ion signalling. As well as a neuroprotective role, there is a body of literature describing how lithium, again *via* the WNT pathway, promotes the differentiation of cells including stem and neuroblastoma cell lines 12–19. It is this particular action of lithium that forms the basis for the screen described here. We selected for gene trap mutated cells that escape from lithium-induced differentiation and continue to proliferate. Our hypothesis was that such cells will have genetic lesions in the signalling and response pathways central to lithium action that may be important for its therapeutic activity. A number of trapped genes were identified in this way adding to our knowledge of the mechanisms of this drug.

## Materials and methods

### Cell culture

SH-SY5Y cells were grown using standard techniques and conditions. In short, cells were cultured with DMEM/F12/Glutamax media (Cat. 31331; Life Technologies, Paisley, UK) supplemented with 10% Foetal Calf Serum (FCS, cat. S1900-500; BioSera, Boussens, France) and 1% penicillin/streptomycin (Sigma-Aldrich, Gillingham, Dorset, UK) in T-25 (Corning, Amsterdam, the Netherlands) and maintained in a 5% CO_2_, 37°C incubator. TryplE Express (Life Technologies) was used to passage cells. During lithium selection, FCS was reduced to 2% to reduce additional, serum-derived proliferation signals.

### Mutagenesis

Gene trap vector pMS1 was acquired from Prof. William Stanford from the Stanford laboratory is at Ottawa Hospital Research Institute, Ottawa, Canada. The plasmid was linearized using *ScaI* restriction enzyme, purified and then introduced into SH-SY5Y cells by nucleofection with Lonza Nucleofector utilizing Reagent V and two different protocols: G04 and A23. In each case, 10^6^ cells were transfected with 2.1 μg of linearized pMS1. Cells were given a 1-week selection period in media supplemented with G418 (250 μg/ml; Sigma-Aldrich). Resistant (productive gene trap integration) cells were seeded into 6-well plates at a density of 150,000 cells per ml.

### Selection for escape from lithium-induced differentiation

Lithium chloride (Sigma-Aldrich) was dissolved in water, filtered and added to the medium. Titration experiments (data not shown) demonstrated that a final concentration of 9.5 mM was the minimal dose required to completely halt wild-type SH-SY5Y proliferation in the presence of 2% FCS. The medium was changed regularly. After 4 weeks, non-proliferating/moderately differentiated cells had mostly died leaving colonies of dividing cells. These were removed from the wells as either individual colonies or as low-complexity mixes of colonies and allowed to expand in T-25 flasks in the absence of lithium and with 10% serum restored.

### Rapid amplification of cDNA ends, cloning and sequencing

RNA was extracted from expanded colonies/colony mixes using a Biometra/Analytik Jena, innuPREP RNA mini kit. cDNA was produced using Roche first-strand cDNA Synthesis kit for RT-PCR (AMV). RACE-PCR was performed to amplify a region of cDNA that spans the splice junction between the endogenous Neo^R^ gene and the 3′ exons of the endogenous trapped gene. The protocol and the primers used in the two-step amplification procedure were all based on information available at the website of Prof. Stanford's laboratory (http://www.cmhd.ca/genetrap/vectors.html). Briefly, the primer used for reverse transcription was 3CDS: 5′ AAG CAG TGG TAA CAA CGC AGA GTA CTT TTT TTT TTT TTT TTT TTT TTT TTT TTT TVN 3′. RACE 1 primers consisted of SD5-P3 (forward) 5′ CGC ATC GCC TTC TAT CGC CTT CTT GAC G 3′ and UPM (reverse), note that UPM is a mixture of 0.4 μM UPL 5′ CTA ATA CGA CTC ACT ATA GGG CAA GCA GTG GTA ACA ACG CAG AGT 3′ and 2 μM UPS 5′ CTA ATA CGA CTC ACT ATA GGG C 3′. The PCR protocol used was a two-step PCR where a second reaction was conducted using primers nested to RACE 1 primers, thus improving specificity. RACE 2 primers composed of 3′RACE1 (forward) 5′ CAA GCG ACG CCC AAC CTG CCA TCA CGA G 3′ and Nest1 (reverse) 5′ AAG CAG TGG TAA CAA CGC AGA GT 3′.

RACE-PCR products were purified and ligated into pGEM1 (Qiagen, Crawley, West Sussex, UK) and chemically competent *Escherichia coli* cells transformed with these ligation products. Antibiotic-resistant bacterial colonies were grown overnight in LB broth and plasmid DNA purified using a kit (Bioline, London, UK). Plasmid DNA was sequenced by Source Bioscience (Nottingham, UK) using primer SD5-P3 (above).

## Results

**Fig. 1:**
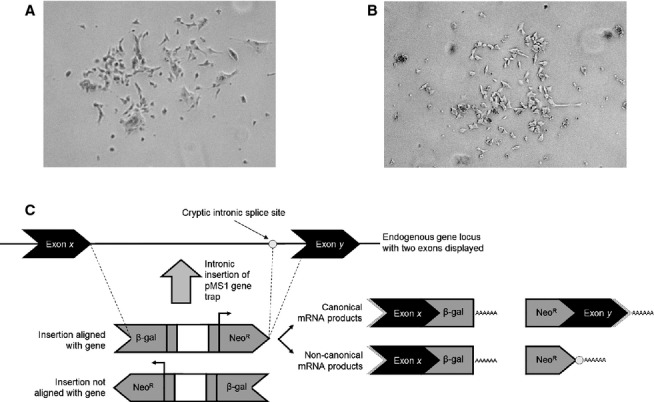
Lithium-resistant cell colonies resulting from gene trap integration events. Two examples of colonies that formed after several weeks of selection in media with 9.5 mM lithium chloride (**A** and **B**). Schematic of observed gene trap events (**C**). An intron between two exons (x and y) of a hypothetical endogenous gene is the site of gene trap integration. Incorrect orientation of the gene trap integration is unlikely to alter splicing of the endogenous gene. Correct orientation of this integration and correct splicing with the surrounding exons produces ‘canonical’ fusion mRNAs. We also observed ‘non-canonical’ splicing to cryptic splice acceptor sites in the intron.

**Fig. 2:**
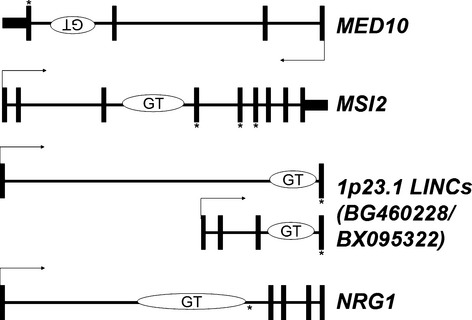
Four examples of gene trap events. Genes are shown in the conventional orientation along their respective chromosome locations and have been compressed/expanded to the same horizontal length for display purposes. Vertical bars represent exons and arrows, transcriptional start sites. Ovals containing GT indicate estimated gene trap integration site. LINCs from chromosome 1p23.1 are alternate splice forms at the same locus. Canonical splicing was observed for the top three events as indicated by the sequencing of endogenous exons (asterisked) in the Neo^R^ fusion mRNAs. The non-canonical *NRG1* integration showed Neo^R^ splicing to intronic sequences.

After a period of 3–4 weeks in 9.5 mM lithium selection a number of growing cell colonies began to emerge from a background of dying cells (Fig. 1A and B). These were eventually removed from each well using tryspin/EDTA, expanded in lithium-free medium supplemented with 10% FCS, and then cloned and sequenced to identify endogenous trapped genes. A list of the ten identified genes is presented in Table 1. Sequencing identified variability in the process of splicing from gene trap sequences to endogenous gene. The gene trap vector pMS1 (Fig. 1C) possesses a splice donor sequence that drives the formation of a hybrid mRNA between the β-actin promoter-driven Neomycin resistance gene in the vector and the 3′ exon(s) and their polyadenylation signal from the endogenous trapped gene. This hybrid mRNA and its protein product should only form (and be capable of providing antibiotic resistance) in instances where the gene trap has inserted in the same orientation as the trapped gene's direction (5′—endogenous gene splice donor—gene trap splice acceptor—gene trap splice donor—endogenous gene splice acceptor—3′) and in an intron relatively close to the 3′ end of the gene. Hence, a ‘canonical’ gene trap mutation event is defined as one in which these conditions have been met and correct fusion transcripts are produced: as illustrated in Figures 1C and 2. Table 1 includes four examples, in the *MSI2*, *MED10* and two long intergenic non-coding (LINC) genes, where we identified such mutation events. In addition, we identified six non-canonical insertional events in which, although clearly intronically inserted, the cloned RACE-PCR products suggested that the Neo^R^ gene had preferentially spliced to a presumed cryptic splice acceptor and polyadenylation sequence within the intron rather than the next endogenous exon's splice genuine acceptor. Two of these indicated that the gene trap sequence had integrated in an orientation against the gene direction. The interpretation of these ‘non-canonical’ events is discussed in detail below.

**Table 1:** Genes identified from lithium-resistant cell colonies and the properties of their gene trap integrations/splice junctions

Trapping event	Gene name	Gene symbol	Accession	Chr.	Gene trap integration site	Orientation	Fusion mRNA type
Canonical	Mediator complex subunit 10	*MED10*	NM_032286	5p15.31	3rd/final intron	With gene	3′ splice to exonic sequence
Canonical	Musashi 2	*MSI2*	NM_138962	17q22	3rd intron	With gene	3′ splice to exonic sequence
Canonical	LINC RNA	N/A	BG211256/BG20390	3q13.32	6th/final intron	With gene	3′ splice to exonic sequence
Canonical	LINC RNA	N/A	BX095322	1p21.3	3rd/final intron	With gene	3′ splice to exonic sequence
Non-canonical	Neuregulin 1 (GGF2 spliceform)	*NRG1*	NM_013962	8p12	1st intron of longest spliceform	With gene	3′ splice to intronic sequence
Non-canonical	Ferric-chelate reductase 1	*FRRS1*	NM_001013660	1p21.2	8th intron of longest spliceform	With gene	3′ splice to intronic sequence
Non-canonical	LINC RNA	*LOC401164*	NR_033869	4q35.2	Final intron	With gene	3′ splice to intronic sequence
Non-canonical	ATP/GTP binding protein-like 4	*AGBL4 (CCP6)*	NM_032785	1p33	3rd intron	With gene	3′ splice to intronic sequence
Non-canonical	LINC RNA	N/A	BQ437861	5q23.2	1st intron	Against gene	3′ splice to intronic sequence
Non-canonical	Latrophilin 2	*LPHN2*	NM_012302	1p31.1	3rd intron	Against gene	3′ splice to intronic sequence

‘Chr.’ indicates chromosomal locus of insertion. ‘Orientation’ indicates whether the gene trap sequences were oriented in the same direction (with gene) or against it.

## Discussion

The molecular actions of lithium have been previously studied by direct assessment of its effect on particular candidate signalling pathways 7, 11 or through its regulation of gene expression 20–24. The gene trap approach applied here is different in that it makes no prior assumptions about the biological activities involved and is based on the functional consequences of gene disruption (cause) rather than the complex transcriptional response to a stimulus (effect).

There are, however, several caveats that should be appreciated. We chose to assess the biological actions of lithium by making use of its previously described effects on the proliferation of neuroblastoma cells 13. Our hypothesis was that the biological pathways and processes that mediate this aspect of lithium's *in vitro* actions would be relevant to its mode of therapeutic action in patients. However, it is quite possible that the list of trapped genes from a different selection strategy that focused on, for example, the neuroprotective action of lithium might differ and be more closely relevant to its mood stabilizer action. The choice of 9.5 mM lithium treatment was based on the minimum concentration that completely inhibited SH-SY5Y cell proliferation. This is in the order of 10-fold greater than therapeutic concentrations typically observed in patients 25. However, we believe we can rule out non-specific toxicity effects as lithium reduced cell proliferation at just 2 mM (data not shown) suggesting that the screen genuinely interrogated gene–lithium interactions relating to proliferation. The coverage of our gene trap screen is also potentially limited by five technical factors: the initial nucleofection efficiency, the inherent preference of this gene trap type for 3′ introns, the inability to detect gene trap mutations that *increase* susceptibility to lithium differentiation, the fact that a proportion of genes lack (or possess very small) introns and the variation in dosage sensitivity effects between genes. The last of these factors is important because a gene trap mutation is a heterozygous loss-of-function event (equivalent to 50% gene product reduction). The study of human genomic copy number variation and the molecular basis of dominant and recessive traits suggests that not all genes that are mechanistically important in any biological system under examination will have detectable/selectable phenotypes through this type of mutation 26. To maximize the potential for a heterozygote gene trap mutation to produce a selectable phenotype we took care to ensure that the lithium concentration employed was the minimum required (a ‘phenotypic threshold’) to elicit a robust differentiation response.

Our choice to study lithium was partly because its biological action is moderately well understood and could thus provide a framework for assessing the performance of the technique. Canonical gene trap insertions disrupted two genes, *MED10* and *MSI2*, that can be directly linked to the WNT/GSK3β/β-Catenin pathway previously implicated in lithium action, whereas none of the identified genes showed straightforward links to inositol metabolism. *MED10* (*Mediator complex subunit 10*) is a component of a multi-protein complex responsible for converting signalling pathways into the transcription of target genes *via* interaction with RNA Polymerase II at gene promoters 27. It is well-established that MED12 and MED13 proteins are Mediator subunits directly translating β-Catenin signalling into a transcriptional response 28, 29 and there is also evidence that MED10 also directly participates in WNT signalling 30. *MSI2* (*Musashi 2*) encodes an RNA-binding protein that is known to regulate the balance between cellular proliferation and differentiation—a function that has been particularly well explored in the context of stem cells 31, 32. MSI2 is also known to be regulated by the WNT pathway *via* signalling through the TCF1 and p21 proteins 33, and a mouse *Msi2* knockdown has been shown to deregulate Wnt pathway target genes 34.

Two canonical insertions were identified in LINC RNA genes on chromosomes 1 and 3. This class of non–protein-coding genes is the subject of much current interest as they are catalogued and their diverse functional properties increasingly understood 35, 36. It is now believed that they can act as direct regulators of mRNA transcripts and chromatin state, and may even aid the formation of large functional protein complexes 37–39. They seem to be particularly important in healthy and disease-related processes in the brain 40–42. LINC RNAs have already been shown to regulate the choice between proliferation and differentiation in stem cells 43. The LINC RNA discovery in this screen suggests that gene trapping can be useful to help assign function to these otherwise hard-to-study molecular species.

As detailed in Table 1, a number of non-canonical insertion events were identified, as defined by the presence of splicing between the Neo^R^ gene and intronic DNA. For example, the non-canonical Neo^R^ splicing event within LOC401164 occurs at the cryptic consensus splice acceptor sequence TCAG/G present in the intronic genomic sequence (see Fig. 1C for a representation of such an event). Some 380 bp downstream of this are two AATAAA sequence motifs—the consensus signal for polyadenylation. Hence, all the requirements for Neo^R^ mRNA/protein expression are met despite this not being a recognized intron–exon boundary. Of the six non-canonical insertion events listed in Table 1, four are aligned in the orientation of the endogenous gene suggesting—by their lithium resistance—that they have nevertheless maintained the ability to disrupt the endogenous gene's transcription: perhaps *via* the gene trap splice acceptor functionality. A gene trap of this type located within *NRG1* (*Neuregulin 1*) is an intriguing finding as there is considerable (albeit not yet concrete) genetic evidence associating variants in this gene with increased risk of schizophrenia and bipolar disorder 44–47. *FRRS1* (*Ferric-chelate reductase 1*, also known as *stromal cell-derived factor 2*) is involved in the uptake, and oxidative state regulation, of iron ions and is known to be expressed in the brain 48. *AGBL4* (*ATP/GTP binding protein-like 4*, also known as *CCP6*, *cytosolic carboxypeptidase 6*) removes glutamate side chains from cytoskeletal proteins—a post-translational modification that has been linked to neurodegeneration 49. Finally, another LINC RNA, *LOC401164*, was identified as an aligned, non-canonical gene trap event.

The two non-aligned gene trap insertions in *LPHN2* (*latrophilin 2*; a regulator of intracellular calcium stores and, hence, neurotransmitter release) and a chromosome 5 LINC RNA may represent false positives because they do not conform to splicing-based disruption models, but they were still identified in cells that continued to proliferate in the presence of lithium. It is possible that more complex and locus-specific endogenous gene deregulation mechanisms could be responsible for their identification in the screen.

It should be pointed out that the screen described in this study has generated a set of candidate genes that will require further study to strengthen their link to clinical lithium action. Specifically, the deleterious action of the gene trap insertions on gene expression should be verified and a process of independent validation carried out (for instance, RNAi knockdown in additional cell lines) to confirm functional involvement. It will be important to determine, in the appropriate cohort, if genes identified in this screen have DNA variants responsible for individual patient differences in response to medication. We have demonstrated that the gene trap approach can usefully address questions of drug action and our findings can be interpreted, in part, as consistent with lithium's effect on the WNT pathway—particularly, the translation of this pathway into nuclear gene expression changes. Gene trap screens targeting laboratory cell lines are still in their infancy compared with RNAi screens or gene trap use in the large-scale production of mutant mouse strains, and yet they have the potential to offer rapid insights into functional biology as well as providing permanently, specifically and consistently genetically modified cell lines for more specific experimentation or small molecule screening 26, 50–52. Future studies should aim to tackle the technological issues that prevent mutation saturation or result in non-canonical splicing events but, overall, the approach is limited only by the ability of researchers to devise novel and appropriate selection/phenotyping criteria.
